# Mann White Slave Law

**Published:** 1916-04

**Authors:** 


**Mann White Slave Law.** The Critic & Guide, March, editorially notes that of 22 persons confined in a penitentiary after conviction under this law, 19 are there "as a result of an absurd perversion of justice or as a result of blackmail." The Bulletin of the Am. Social Hygiene Assn., Jan., states that a systematic process of blackmail has been carried on by the plan of placing prominent men in compromising situations and that a million dollars in hush money has been collected. (Note: While expressly waiving support of vice in any form, we took the ground at the beginning of the discussion of this law that any legislation which passes from the standard of actual damage and overt violence to that of pure morality, using the term morality in its broadest sense, is liable to abuse and impossible of execution except under circumstances that favors blackmail.—Ed.)

